# ﻿First occurrence of the little-known genus *Noteriades* (Hymenoptera, Megachilidae) from Vietnam: discovery of a new species and a key to the Southeast Asian fauna

**DOI:** 10.3897/zookeys.1102.82466

**Published:** 2022-05-20

**Authors:** Ngat Thi Tran, Michael S. Engel, Lam Xuan Truong, Lien Thi Phuong Nguyen

**Affiliations:** 1 Graduate University of Science and Technology, Vietnam Academy of Science and Technology, 18 Hoang Quoc Viet Road, Nghia Do, Cau Giay, Hanoi, Vietnam Graduate University of Science and Technology Hanoi Vietnam; 2 Institute of Ecology and Biological Resources, Vietnam Academy of Science and Technology, 18 Hoang Quoc Viet Road, Nghia Do, Cau Giay, Hanoi, Vietnam Institute of Ecology and Biological Resources Hanoi Vietnam; 3 Division of Entomology, Natural History Museum, and Department of Ecology and Evolutionary Biology, 1501 Crestline Drive – Suite 140, University of Kansas, Lawrence, Kansas 66045-4415, USA University of Kansas Lawrence United States of America

**Keywords:** Anthophila, Apoidea, Megachilinae, morphology, resin bees, taxonomy

## Abstract

The little-known megachiline genus *Noteriades* Cockerell, 1931 is recorded from Vietnam for the first time. A new species, *Noteriadeshangkia* Tran, Engel & Nguyen **sp. nov.** is described and figured based on a series of females collected from the provinces of the northern and central highlands of Vietnam. The genus is briefly discussed and a new subtribe is established, Noteriadina Engel, Tran & Nguyen **subtrib. nov.** of Megachilini. Lastly, an identification key and distribution map are provided for those species occurring in Southeast Asia.

## Abstract (vt)

Lần đầu tiên ở Việt Nam, một giống ong ít được biến đến có tên *Noteriades* Cockerell, 1931 đã được phát hiện. Trong giống này, *Noteriadeshangkia* Tran, Engel & Nguyen, **sp. nov.** được mô tả là loài mới cho khoa học cùng hình ảnh minh họa dựa trên các cá thể cái thu thập được ở một số tỉnh miền Bắc và Tây Nguyên của Việt Nam. Từ việc thảo luận về kết quả nghiên cứu của giống, một phân tộc mới, Noteriadina Engel, Tran & Nguyen, **subtrib. nov.** đã được thiết lập. Khóa định loại đến loài và bản đồ phân bố của các loài thuộc giống này ở Đông Nam châu Á cũng được đưa ra.

## ﻿Introduction

The megachiline bee genus *Noteriades* Cockerell is a seemingly relict genus, with comparatively few species occurring in both temperate and tropical regions of sub-Saharan Africa and southern Asia (Michener 2007). Traditionally, the genus was classified in the tribe Osmiini and among the *Heriades*-group of genera owing to its distinctly hoplitiform body habitus and size (Michener 2007), but as originally hypothesized by Griswold (1985)*Noteriades* has recently been recovered as the sister group to Megachilini (Praz et al. 2008; Gonzalez et al. 2012, 2019). Today, the genus is classified as the extant sister group to all other Megachilini (Gonzalez et al. 2019), a noteworthy position as the tribe otherwise includes the famous leaf-cutter and resin bees of the genus *Megachile* Latreille and its relatives (Michener 2007; Gonzalez et al. 2019). Unfortunately, nothing is known of the biology of any species of *Noteriades*, the discovery of which melittologists are encouraged to seek.

Griswold and Gonzalez (2011) provided a provisional list of species for *Noteriades*, including 16 species, with most occurring in Africa. In Southeast Asia, there currently occur three species: *Noteriadesjenniferae* Griswold & Gonzalez, 2011; *N.pulchripes* (Cameron, 1897); and *N.spinosus* Griswold & Gonzalez, 2011 which have been found in India, Thailand, and Myanmar. A further four species, all described from northern India (Gupta 1993), are of uncertain generic affiliation, are poorly documented, and in need of revision. Indeed, there is reason to believe they are misidentified and belong to another genus of Osmiini (perhaps even as synonyms of other species), as evidenced by the tridentate mandibles and seeming absence of a mediolongitudinal carina on the clypeus (Gupta 1993). For the moment, these species are best considered as *nomina dubia*.

Here, we report the genus *Noteriades* for the first time from Vietnam, represented by a new species. We provide a description and figures for the species, and an identification key and distributional map for all Southeast Asian species. In addition, given the considerable morphological disparity between *Noteriades* and the remainder of Megachilini, we establish a new subtribe for the genus.

## ﻿Materials and methods

Specimens examined in the present study are deposited in the hymenopteran collections of the Institute of Ecology and Biological Resources (**IEBR**), Hanoi, Vietnam and the Division of Entomology (SEMC), University of Kansas Natural History Museum, Lawrence, Kansas, USA (**SEMC**). Adult morphological and color characters were examined with a Nikon SMZ745 stereomicroscope, while images were photographed with a Nikon SMZ800N digital stereomicroscope, and with an ILCE-5000L/WAP2 digital camera attached to the stereomicroscope. Stacked focus images were prepared using with Helicon Focus 7. Finally, all files were processed with Adobe Photoshop CS6. The morphological terminology used in the description follows Engel (2001) and Michener (2007), with certain body metrics following those of Niu et al. (2004): specifically, **body length**: measured from the base of the antennal torulus to metasomal apex (in dorsal view), **head length**: measured from the medioapical margin of the clypeus to the upper margin of the vertex (in facial view), **head width**: measured at the widest point of the head across the compound eyes (in facial view), **eye width**: the greatest width of the compound eye (in profile), **genal width**: the greatest width of the gena (in profile), **mesosomal width**: measured between the outer rims of the tegulae (in dorsal view).

The abbreviations F, S, and T (followed by Arabic or Roman numerals) refer to numbered flagellomeres, metasomal sterna, and metasomal terga, respectively. The classification of Megachilini adopted herein is that of the extensive morphological and molecular treatment of Gonzalez et al. (2019).

## ﻿Systematics

### ﻿Tribe Megachilini Latreille, 1802

#### ﻿ Noteriadina

Taxon classificationAnimaliaHymenopteraMegachilidae

Engel, Tran & Nguyen subtrib. nov.

55D3BAE4-09B7-539F-81EA-0AB46D953077

http://zoobank.org/AA0BBF9D-5715-4ADF-895C-0776BF3C9F24

##### Type genus.

*Noteriades* Cockerell, 1931.

##### Diagnosis.

Small to modest-sized (4.5–10.2 mm), non-metallic, hoplitiform bees lacking integumental maculation; mandible of female quadridentate, without differentiated cutting edges, mandible of male bidentate; malar space linear; clypeus slightly projecting over clypeal-labral articulation; clypeus and often supraclypeal area with mediolongitudinal carina; paraocular area with dense appressed pubescence; preoccipital carina complete. Pronotum not enlarged nor surrounding mesoscutum anteriorly; pronotal lobe and omaulus carinate, with defined omaular surface; mesoscutellum flat, carinate posteriorly, overhanging metanotum (scarcely so in *N.pulchripes*); propodeum wholly vertical, without basal subhorizontal zone; outer surfaces of pro- and mesotibiae apically with an acute angle and distinct notch anteriorly, therefore appearing bispinose in apical view; arolia present on all legs in both sexes (absent in Megachilina except *Matangapis* Baker & Engel and *Heriadopsis* Cockerell). Metasomal tergum I carinate dorsally at angle between anterior- and dorsal-facing surfaces; tergum VI of female nearly vertical except for apical flange-like hyaline margin, without preapical carina, tergum VI of male without preapical carina (present in Megachilina); terga V and VI of male strongly curved ventrally (only terga I–IV visible in dorsal view), covering tergum VII and sterna III–VI (no so in Megachilina); sternum I of male produced over its apical margin subapically, forming double carina (not so in Megachilina); volsella distinct, with well-developed digitus and cuspis, with heavily sclerotized denticles resembling those of short-tongued bee families and *Pararhophites* Friese. Refer to Gonzalez et al. (2019) for the supraspecific classification of Megachilini.

#### ﻿Genus Noteriades

Taxon classificationAnimaliaHymenopteraMegachilidae

Cockerell, 1931

E8B2897A-63DF-5049-85B4-D03B9CE081EF

Heriades (Noteriades) Cockerell, 1931: 332. Type species: Megachiletricarinata Bingham, 1903, by original designation.

##### Diagnosis.

As for the subtribe (*vide supra*).

#### ﻿ Noteriades hangkia

Taxon classificationAnimaliaHymenopteraMegachilidae

Tran, Engel & Nguyen sp. nov.

C2F84703-7924-54E0-8255-1A47C7DA1CB4

http://zoobank.org/6F3809A1-E0A4-4188-9406-FF430B21F594

Figs 1–2 , 3–6

##### Type material.

***Holotype*.** Vietnam: ♀, Hoà Binh, Mai Chàu, Hang Kia, alt. 1200 m, 12.vi.2008 [12 June 2008], Liên Thị Phương Nguyễn, Phong Huy Phạm leg.” [IEBR].

***Paratypes*.** Vietnam: 1♀, same data as holotype [SEMC]; 1♀, Tuyên Quang, Hàm Yên, Yên Thuận, Cao Đường, Cham Chu NR, 22°20'16.4"N, 103°51'09.4"E, alt. 670 m, 16.v.2019 [16 May 2019], Cường Quang Nguyễn, Liên Thị Phương Nguyễn leg.; 2♀♀, Kon Tum, Sa Thầy, Chư Mom Ray NP, 14°47'24.5"N, 107°59'46.5"E, alt. 729 m, 25.iv.2016 [25 April 2016], Liên Thị Phương Nguyễn, Đắc Đại Nguyễn, Ngát Thị Trần leg.; 6♀♀, Kon Tum, Sa Thầy, Chư Mom Ray NP, Ro Koi RS, 14°27'25"N, 107°36'22"E, alt. 267 m, 25.iv.2022 [25 April 2022], Liên Thị Phương Nguyễn, Ngát Thị Trần leg. [IEBR].

##### Diagnosis.

The female of this species is most similar to that of *N.jenniferae* as both have the apical margin of the clypeus crenulate, the mediolongitudinal carina distinctly extends onto the supraclypeal area; and the apical margin of the mesoscutellum is rounded, without apicolateral spines. The new species can be distinguished in the female from latter species by the following characters: F1 shorter than F2 (F1 about as long as F2 in *N.jenniferae*); the rim of the antennal torulus mesodorsally extended into a short lamellate tubercle (the rim of the antennal torulus unmodified and not mesodorsally extended in *N.jenniferae*); mesosoma approximately as long as broad (mesosoma longer than broad in *N.jenniferae*). In addition, the new species differs from both *N.jenniferae* and *N.spinosus* by the generally shiny face and mesoscutum, which is matte in the latter two species.

##### Description.

♀: Body length 8.0–8.5 mm (holotype = 8.5 mm), forewing length 5.5–6.0 mm (holotype = 6.0 mm).

***Structure*.** Head slightly broader than long, approximately 1.1× as broad as long (Fig. 3). Compound eyes subparallel, 2.5× as long as broad, about 1.3× genal width. Mandible quadridentate, without differentiated cutting edges. Clypeus slightly convex on basal half, 1.8× as broad as long, apical margin crenulate, mediolongitudinal carina distinct, extending onto supraclypeal area (Fig. 3). Supraclypeal area slightly convex. Juxtantennal carina absent. Interantennal distance about 1.6× median ocellar diameter; antennal torulus with rim mesodorsally extended into short lamellate tubercle (Fig. 4), scape about 2.6× as long as broad, pedicel approximately 1.5× as long as broad and about 2× F1 in length, F1 broader than long and about 0.75× F2 in length, F3–F9 subequal in length, F10 longest flagellomere, longer than broad. Mesosoma approximately as long as broad (Fig. 5); mesoscutum without spine or sharp angle apicolaterally; mesoscutellum apical margin rounded, without apicolateral spines (Fig. 5). Forewing prestigma about as long as 1Rs; pterostigma longer than broad (Fig. 6), margin inside marginal cell convex; marginal cell apex broadly rounded and minutely appendiculate, offset from anterior wing margin; 1Rs not perfectly aligned with 1M, 1M weakly arched anteriorly, distad 1cu-a by about 2–2.5× vein width, thus forming exceedingly short 2M+Cu; Rs+M faintly sinuate; two submarginal cells (i.e., 1rs-m absent), first submarginal cell broader than second submarginal cell; 1Rs straight, about as long as r-rs; r-rs arising at pterostigmal midlength; 1m-cu strongly distad 1Rs; 2m-cu basad 2rs-m (in some paratypes, 2m-cu confluent with 2rs-m), 2rs-m strongly arched. Pretarsal claws with arolia on all legs. Anterior-facing surface of T1 strongly concave (Fig. 2) and dorsally rimmed by strong carina. Pygidial plate absent.

**Figures 1, 2.**
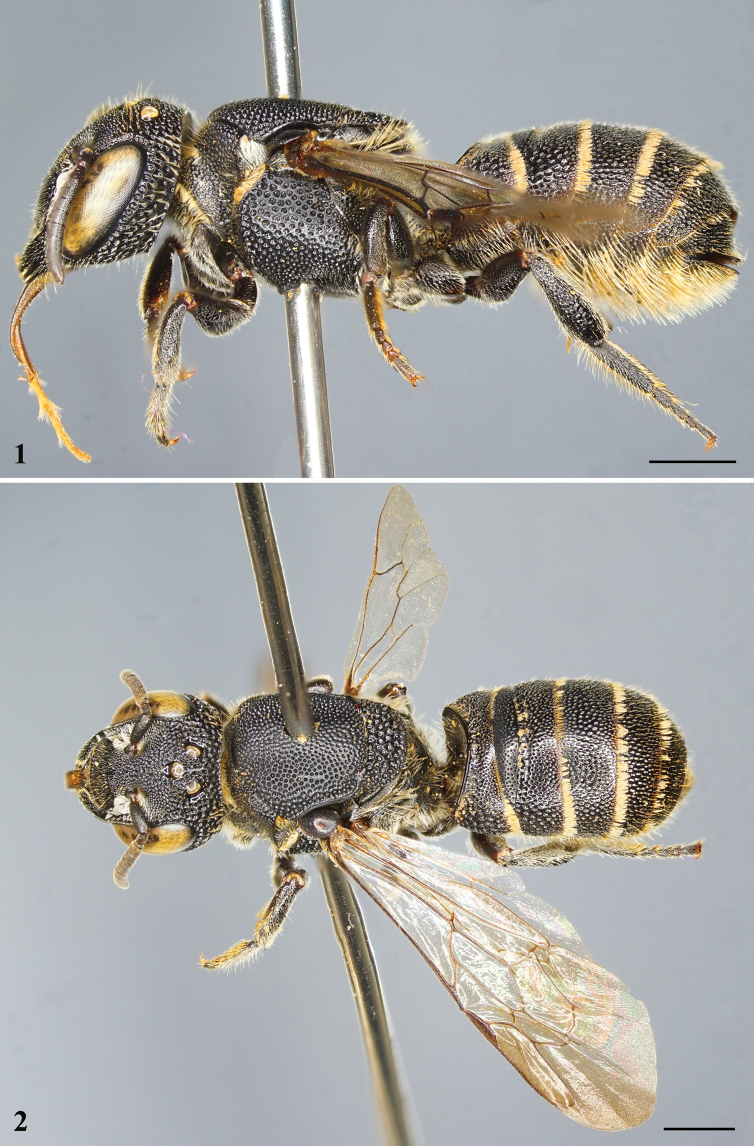
*Noteriadeshangkia* Tran, Engel & Nguyen, sp. nov., holotype, female **1** habitus in lateral view **2** habitus in dorsal view. Scale bars: 1 mm.

**Figures 3–6.**
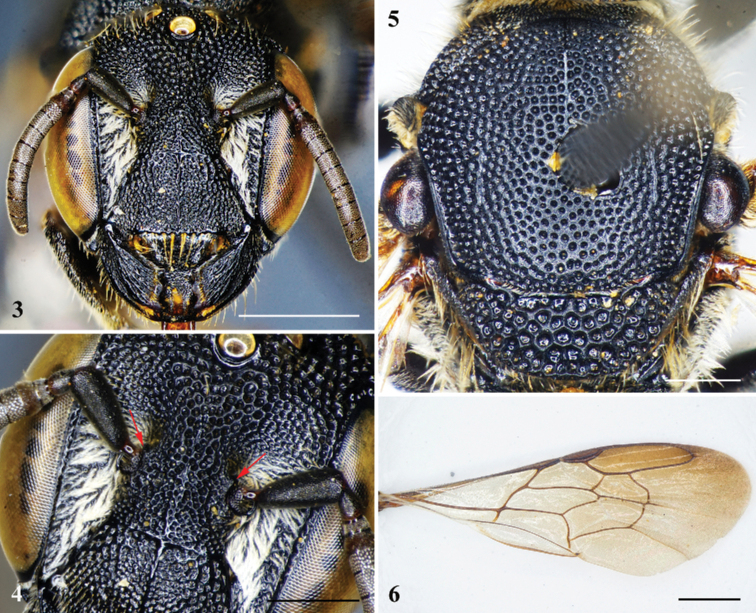
*Noteriadeshangkia* Tran, Engel & Nguyen, sp. nov., holotype, female **3** head in facial view **4** head in anterolateral oblique view showing lamellate extensions from antennal torular rims (red arrows) **5** mesosoma in dorsal view **6** forewing, dorsal view. Scale bars: 1 mm (**3, 5–6**); 0.5 mm (**4**).

***Sculpturing and texture*.** Integument of head and mesosoma generally shiny. Mandible and labrum irregularly punctate, punctures slightly coarser on mandible basally, outer ridges smooth and shiny. Clypeus with contiguous punctures of unequal sizes, puncture sizes laterally and along base larger than on remainder of surface (Fig. 3). Supraclypeal area with contiguous punctures, puncture sizes as on base and sides of clypeus. Frons with contiguous, large, coarse punctures (Fig. 4). Punctures on vertex and gena larger and coarser than on frons, punctures largest on gena. Pronotum with dense coarse punctures, punctures smaller than those on mesoscutum. Mesoscutum with largely contiguous, coarse punctures of subequal sizes, punctures of disc more separated, separated by 0.2–0.5× a puncture width, integument between faintly imbricate; tegula imbricate and impunctate; axilla with contiguous coarse punctures, punctures about 0.5× size of those on remainder of mesoscutellum; mesoscutellum with contiguous, large, coarse punctures, punctures much coarser than those on gena, almost appearing areolate (Fig. 5). Mesepisternum with large, coarse punctures on upper half, separated by about 0.3–0.5× a puncture width, lower half with smaller, denser punctures, such punctures nearly contiguous (Fig. 1). Anterior-facing surface of T1 smooth, shining, impunctate; dorsal-facing surface of T1 and remaining metasomal terga with nearly contiguous, smaller punctures resembling those of frons, except laterally punctures noticeably larger, coarser, and contiguous; S1 with small, sparse punctures; S2–S6 with small, dense punctures, except marginal zones impunctate.

***Color*.** Body black except antenna beneath, tegula, tarsi, and metasomal sterna apical margins dark reddish brown. Wings light brown with faint green mixed coppery highlights in ventral view, membrane of marginal cell and apex darker brown than remainder of remigium and lighter in radial and first cubital cells; veins brown to dark brown, prestigma and pterostigma dark brown.

***Pubescence*.** Paraocular area from epistomal sulcus to slightly above antennal toruli with long, dense, plumose, appressed, white setae, some setae tinged yellowish (Fig. 1). Apical margin of clypeus with sparse, erect, yellow to tawny yellow setae. Outer surfaces of mandible and labrum with minute, erect, yellow to tawny yellow setae, particularly numerous in grooves of mandible. Dorsal surface of pronotal collar, pronotal lobe, lateral surfaces of coxae with short, minutely branched, yellow to yellow tawny setae, those more dorsally on pronotal lobe off white; metanotum and propodeum with longer, erect, minutely branched, yellow setae. Retrolateral surfaces of tarsi with dense, erect, yellowish setae. Metasomal T1–T4 with apical fasciae composed of yellowish plumose setae, medially interrupted on T1–T2, interruption with weak vibrissae composed of scattered, minute, simple setae on T1 (Fig. 2), otherwise discs with scattered short, suberect, yellowish, simple setae, such setae more prominent laterally and progressively longer on T4–T5; S2–S6 with yellowish scopal setae (Fig. 1).

♂: *Latet*.

##### Etymology.

The specific epithet is a toponym for the locality at which the holotype was collected, the Hang Kia commune in Hoa Binh Province. The name is treated as a noun in apposition.

##### Comments.

**Figure 7.**
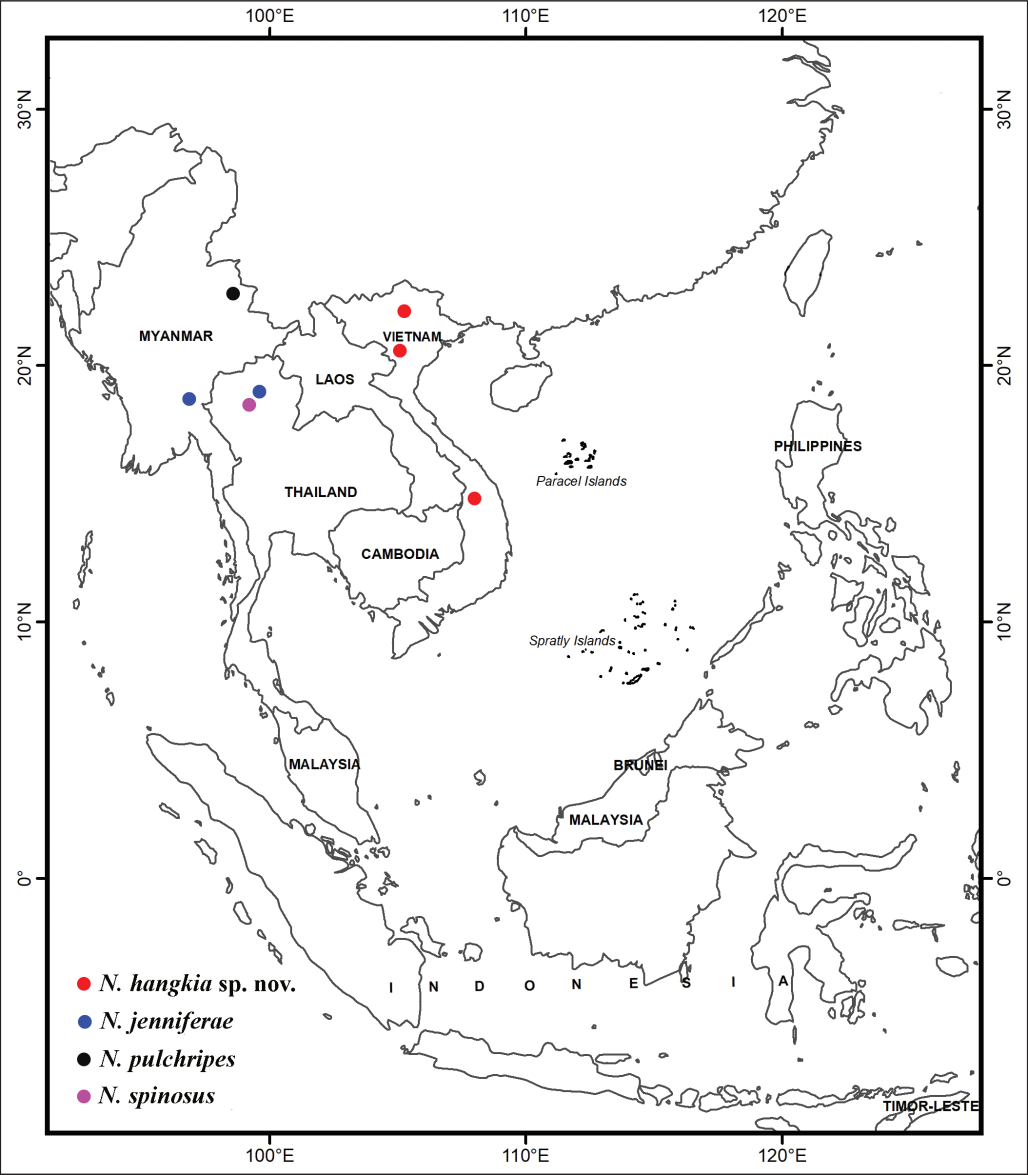
Distribution map of Southeast Asian species of *Noteriades* Cockerell (Megachilinae: Megachilini: Noteriadina).

The discovery of *N.hangkia* in the northern and central highlands of Vietnam extends the distribution of the genus *Noteriades* in Southeast Asia (Fig. 7). In fact, it is likely that the genus shall be found eventually in Laos, Cambodia, and southernmost China.

### ﻿Key to the species of *Noteriades* occurring in Southeast Asia

Characters for the key were extracted from the original descriptions of the species (Cameron 1897; Griswold and Gonzalez 2011).

**Table:** 

1	Mesoscutellum with short apicolateral spines	**2**
–	Mesoscutellum rounded apically, without spines laterally	**3**
2	Mesoscutellar spines broadly triangular, not curved mesally; apical fascia of silvery setae on tergum II not interrupted medially	***N.pulchripes* (Cameron, 1897)**
–	Mesoscutellar spines curved mesally; apical fascia of white setae on tergum II interrupted medially	***N.spinosus* Griswold & Gonzalez, 2011**
3	F1 shorter than F2; rim of antennal torulus mesodorsally extended to form short lamellate tubercle; mesosoma approximately as long as broad; face and mesoscutum generally shiny	***N.hangkia* Tran, Engel & Nguyen, sp. nov.**
–	F1 about as long as F2; rim of antennal torulus unmodified, without mesodorsal lamellate extension; mesosoma longer than broad; face and mesoscutum generally matte	***N.jenniferae* Griswold & Gonzalez, 2011**

## Supplementary Material

XML Treatment for Noteriadina

XML Treatment for Noteriades

XML Treatment for Noteriades hangkia
